# Medicare claims–based evaluation of atrial fibrillation within implantable cardiac monitor patients with cryptogenic stroke

**DOI:** 10.1007/s10840-023-01629-2

**Published:** 2023-08-29

**Authors:** Steven Mullane, Camden Harrell, Nikolaos Tsitiridis, Gaurav Upadhyay, Jim W. Cheung, David Hayes

**Affiliations:** 1 BIOTRONIK, Inc., 6024 Jean Road, Lake Oswego, OR 97035 USA; 2 BIOTRONIK SE & Co. KG, Woermannkehre 1, 12359 Berlin, Germany; 3 Department of Medicine, Section of Cardiology, Center for Arrhythmia Care, The University of Chicago Medicine, Pritzker School of Medicine, Chicago, IL USA; 4 Department of Medicine, Weill Cornell Medicine–New York Presbyterian Hospital, New York, NY USA

## Background

In patients with cryptogenic stroke (CS), the risk of recurrent stroke is high, making early detection and treatment essential [1]. Implantable cardiac monitors (ICMs) are an important diagnostic tool that can detect arrhythmias that may have gone undetected during routine electrocardiogram monitoring (ECG) and are increasingly being used to diagnose arrhythmias in patients with CS [2]. Studies have shown that the use of ICMs improves the detection rate of atrial fibrillation (AF) compared to standard ECG monitoring [2].

To help identify patients who are at increased risk of recurrent stroke and may benefit from anticoagulation therapy, the European Society of Cardiology (ESC) guidelines for the management of AF recommend the use of ICMs in patients with CS to detect AF [3]. Additionally, the American Heart Association/American Stroke Association (AHA/ASA) guidelines have long-term cardiac monitoring for patients with CS as a Class IIa recommendation when external monitoring is inconclusive [4].

The UK’s National Institute for Health and Care Excellence (NICE) guidance, however, does not currently recommend the use of certain ICMs to help detect AF after CS due to lack of research [5]. Therefore, this analysis aims to further evaluate diagnostic yield of AF for CS patients with a BIOTRONIK ICM.

## Methods

### Data sources and patient identification

This analysis utilized the CERTITUDE real-world database, which has been described previously [6], to retrospectively investigate Medicare Fee-For-Service (FFS) beneficiary patients with BIOMONITOR III/IIIm (BIOMONITOR) and indication related to CS with no prior history of AF. Indication of CS was identified from BIOTRONIK Home Monitoring (HM) data. Patients with prior AF diagnosis identified in Medicare FFS administrative claims data at time of implant were excluded from the analysis.

### Identification of atrial fibrillation

The primary outcome of interest was the diagnosis of AF following the implantation of BIOMONITOR. To identify cases of AF, non-overlapping Medicare FFS inpatient, outpatient, or carrier claims were examined for primary or secondary diagnosis codes indicative of AF using claims as established in Center for Medicare and Medicaid Services (CMS) Chronic Conditions Data Warehouse validated algorithms [7]. Patients with such codes were considered to have a diagnosis of AF. Additionally, daily maximum device-detected AF burden was also evaluated from transmitted HM data.

### Comparison to CRYSTAL AF trial 

The results obtained from the study were compared to the historical reported results of the CRYSTAL AF trial [2]. The comparison aimed to assess the agreement between the real-world data from the CERTITUDE database and the findings from this clinical trial. The proportion of patients diagnosed with AF following implantation was calculated based on the patients identified as having AF in Medicare claims data. The comparison to the CRYSTAL AF study was performed using binomial proportion test to determine the level of agreement.

## Results

**Fig. 1:**
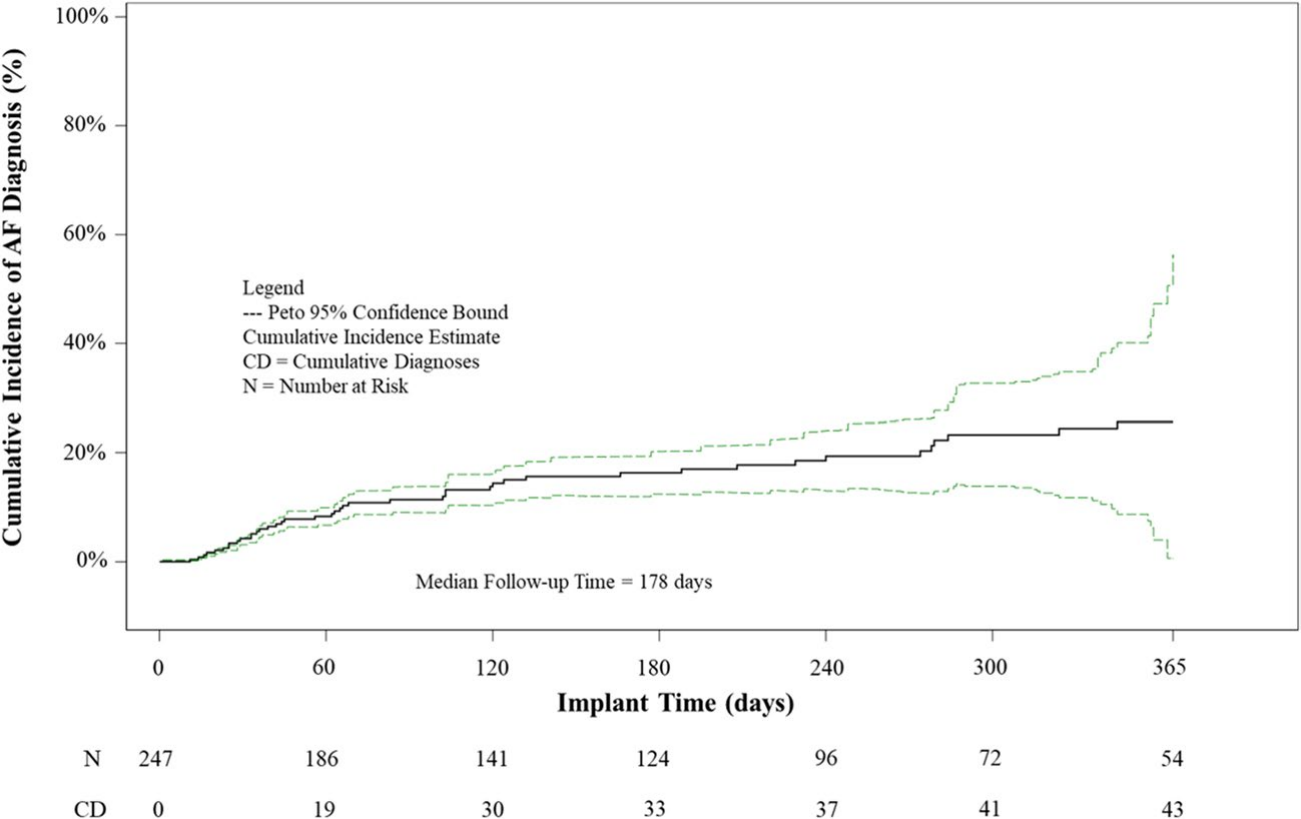
Cumulative incidence of AF diagnosis by 12 months

A total of 247 eligible patients were identified in the CERTITUDE database meeting eligibility requirements. By 12 months, 17.4% (43 of 247) patients had been diagnosed with AF as identified in CMS claims (95% CI of 12.9%, 22.7%) and an incidence rate of 0.341 events/subject-year with a total follow-up time of 125.9 years. Cumulative incidence of AF is shown in Fig. 1. From HM device data, the maximum atrial burden percentage of the 43 patients diagnosed with AF was 18.4% + / − 26.7%.

### Comparison to CRYSTAL AF trial

The BIOMONITOR cohort demonstrated a significantly higher proportion of AF diagnosis at 12 months compared to the 1.9% (4/213) of patients with AF diagnosis in the non-ICM cohort from the CRYSTAL AF trial (*p* < 0.001). No significant difference (*p* = 0.0826) in proportion of AF diagnosis was found between BIOMONITOR and the CRYSTAL AF trial ICM cohort of 13.6% (29/213) patients with AF diagnosis at 12 months.

## Discussion

In this retrospective real-world data analysis, we found 17.4% of CS patients were diagnosed with AF within 12 months following ICM implant. The high prevalence of AF in patients with CS has been well-documented in previous studies, such as the CRYSTAL AF trial, which was a landmark study that demonstrated the value of ICMs in this patient population [2]. Our real-world data findings align with the results of this study, further supporting the effectiveness of ICMs, such as the BIOMONITOR, in identifying AF in CS patients.

While not evaluated in this analysis, patient characteristics and comorbidities may influence the rate of AF diagnosis. Future studies could explore the impact of patient characteristics on AF detection rates to better understand the patient populations that may benefit the most from ICM monitoring.

Overall, the use of ICMs in patients with CS is a valuable diagnostic tool and has gained recognition in clinical practice and by ESC and AHA/ASA [4, 5] to help identify underlying arrhythmias that may have gone undiagnosed and help guide appropriate therapy to reduce the risk of recurrent stroke. Our analysis provides further evidence to support these guidelines.

### Limitations

This analysis relied on patients with Medicare FFS coverage, which may not be fully representative of the general population. To minimize possibility of false-positive detections from ICMs, we focused on clinical diagnosis of AF within CMS claims data instead of the device-detected atrial burden or atrial episodes, but the accuracy of the diagnosis of AF in claims is subject to potential coding errors.

## Conclusion

Our study highlights the importance of ICMs in the diagnosis of AF in patients with CS indication. The detection rate of 17.4% aligns with prior studies, including the landmark CRYSTAL AF trial, and underscores the clinical significance of ICMs, such as the BIOMONITOR, in this patient population. By identifying AF, physicians can initiate appropriate management strategies, including anticoagulation therapy, to reduce the risk of recurrent stroke.

## Data Availability

Due to Data Use Agreement with CMS, supporting data is not available.
